# An Alternative Technique in Laparoscopic Cholecystectomies: Removal Without a Specimen Bag

**DOI:** 10.7759/cureus.7655

**Published:** 2020-04-12

**Authors:** Matthew Heard, Jessica Rehrig, Donato Colavita

**Affiliations:** 1 General Surgery, College of Osteopathic Medicine, University of New England, Biddeford, USA; 2 Osteopathic Medicine, University of New England, Biddeford, USA; 3 Neurology, North Shore University Hospital, Long Island, USA; 4 General Surgery, St. Michael's Medical Center, Newark, USA; 5 General Surgery (Emeritus), Saint Barnabas Medical Center, Livingston, USA

**Keywords:** laparoscopic cholecystectomy, specimen bag, laparoscopic technique, gallbladder removal, surgical technique

## Abstract

The use of specimen retrieval bags in elective laparoscopic cholecystectomies remains a customary practice intended to reduce surgical site infections. The lack of supporting evidence suggests that specimen bags may not be necessary. Thus, we present an alternative approach without the use of disposable instruments to reduce excessive healthcare costs while maintaining comparable surgical outcomes.

## Introduction

Cholecystectomy is one of the most common abdominal surgeries in the United States, with approximately 850,000 cases performed annually [1]. The first laparoscopic cholecystectomy was performed in 1987 and received widespread approval from the general surgery community for its reduced cost compared to open procedures, decreased hospital length of stay, and increased patient satisfaction. Today, 90% of cholecystectomies in the United States are performed laparoscopically and it is considered the “gold standard” for elective cholecystectomy [1,2].

The Society of American Gastrointestinal and Endoscopic Surgeons (SAGES) guidelines leave the use of a specimen retrieval bag in elective cholecystectomies up to the discretion of the operating surgeon [3]. Some surgeons reason that evacuating the gallbladder in a bag will decrease surgical site infection (SSI) by eliminating the contact between gallbladder and the wound, but research has shown no significant benefit in this population in regards to decreased risk of SSI [4]. 

As the financial burden of healthcare continues to increase, surgeons are pressured to reduce their operating costs. Given the frequency of this elective procedure, considerations should be given to whether specimen bags are a necessary expenditure. We present an alternative approach to gallbladder removal in laparoscopic cholecystectomy without the need for a specimen retrieval bag.

## Technical report

The patient is prepped and draped in the usual manner. Abdominal access is achieved with three ports: a 10-12 mm port in the umbilicus and two 5 mm ports in the right subcostal region and subxiphoid area. A 5 mm port may be utilized in the anterior axillary line, right flank or right upper quadrant, depending on body habitus, if more adequate exposure is needed. Once inside the abdominal cavity, the gallbladder is dissected away from any vascular or biliary attachments, clipped, and excised in the usual fashion.

**Figure 1:**
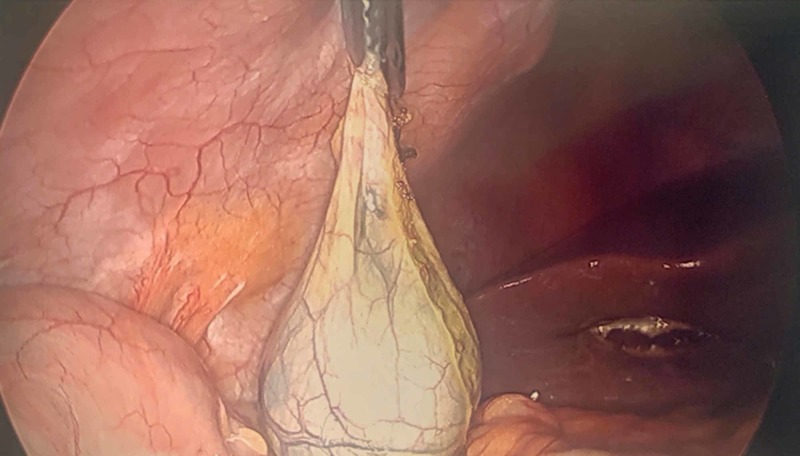
Removal of gallbladder without a specimen bag. The neck or cystic duct is grasped with an atraumatic grasper. A toothed grasper may be used if necessary.

To begin extraction from the abdominal cavity, a 5 mm atraumatic, or toothed, grasper secures the gallbladder near the cystic duct or neck of the gallbladder (Figure 1). The right upper quadrant port provides access to appropriately maneuver the instrument for removal.

**Figure 2:**
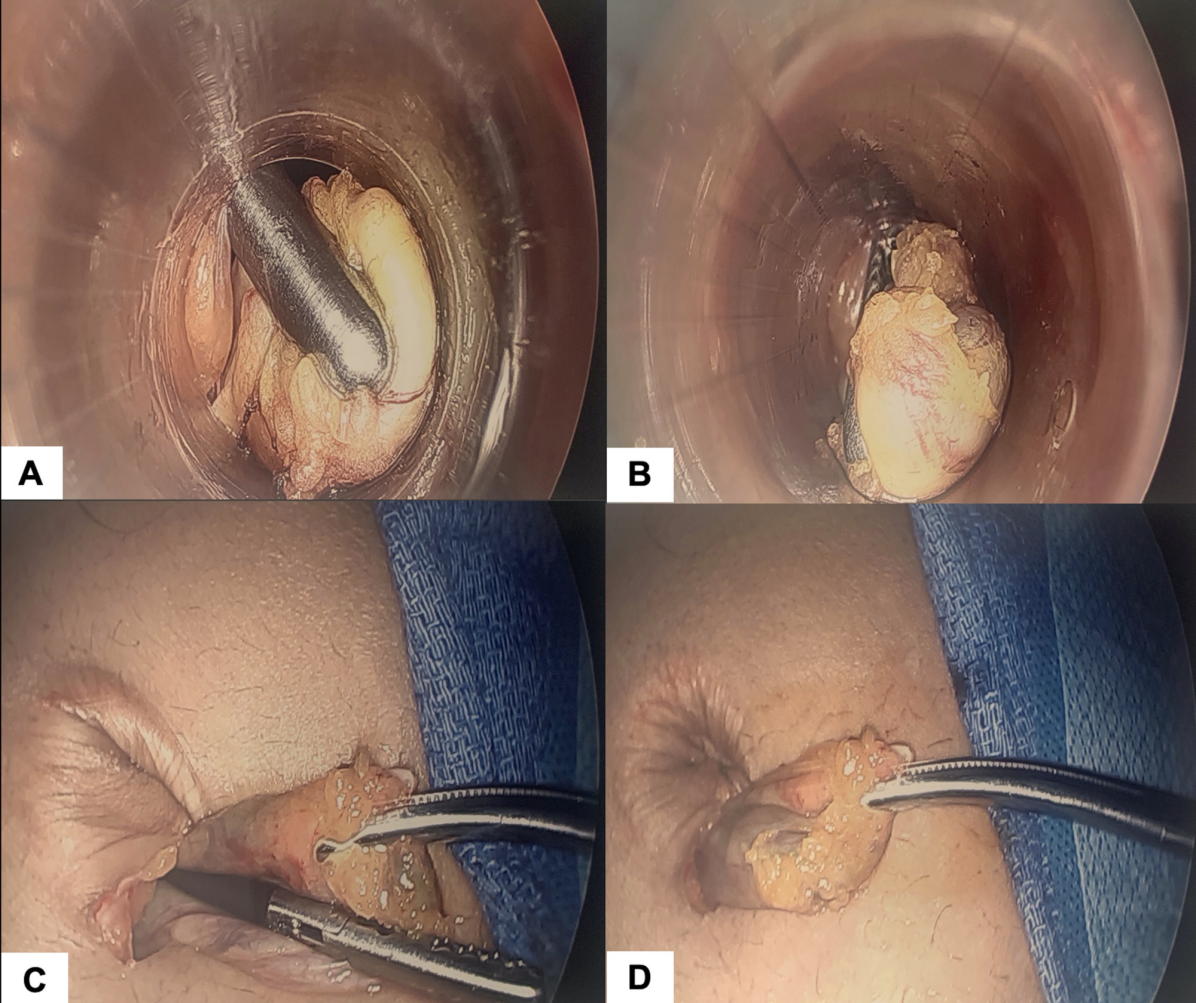
Removal of gallbladder without a specimen bag (A) The grasper holding the gallbladder is placed into the internal opening of the umbilical port. (B) Pressure is applied to the gallbladder and the port. The port is then removed. (C) After removal of the port, the presenting part of the gallbladder is outside of the umbilical incision. The internal grasper can now release the gallbladder after it is grasped on the outside by a Kelly clamp. (D) The gallbladder can be removed immediately as described or it can be decompressed with an incision and evacuation of contents.

Next, the tip of the grasper holding the cystic duct (or neck of gallbladder) is aimed toward and placed into the internal opening of the umbilical port (Figure 2A). The port is removed with pressure applied to the grasper and umbilical port (Figure 2B). The portion of gallbladder held by the internal grasper is now at the level of the umbilical incision site (Figure 2C). The external portion of gallbladder is grasped with a Kelly clamp. The internal grasper can then be released from the gallbladder (Figure 2D).

Several techniques can be utilized when removing the gallbladder, depending on the size of stones and/or organ:

1. Small calculi not requiring gallbladder decompression can be removed with a gentle “back and forth” motion.

2. For innumerable or large calculi, an incision in the extracorporeal gallbladder wall allows contents to be evacuated by suction and a sponge holder.

3. The fascial opening can be enlarged for removal of large, unbreakable stones.

4. If the integrity of the gallbladder wall is breached during mobilization, and content has spilled, this technique is still applicable. A retrieval device is only needed if leaked stones are unable to be retrieved with a sponge holder.

If intraoperative decompression of the fundus occurs, the surgeon should remove the gallbladder by grasping the fundus instead of the neck or cystic duct. After the gallbladder exits the abdominal cavity through the incision site, the operation is completed in its usual fashion.

## Discussion

Technique indications

Our technique has been performed approximately 2,500 times over a span of 15 years, being utilized in 200-300 cases each year by a single surgeon. It has proven to serve as a viable technique in elective laparoscopic removal of the gallbladder. The surgeon has yet to experience an SSI at the umbilical port site in the time since the technique was first performed.

Elective cholecystectomy with a thin walled gallbladder and acute cholecystitis with a relatively thin compressible wall are absolute indications for this technique. Contraindications to this technique include open cholecystectomy, gallbladder with a thick non-compressible wall, and perforated or infected gallbladder. In these cases, the risks of seeding the abdomen with calculi or infection outweigh the benefits of this technique.

Techniques in current literature

The majority of surgical textbooks reference specimen removal but fail to describe a comprehensive technique. The SAGES Manual states the gallbladder should be extracted from the umbilical port “by holding region of cystic duct with a large grasper” [5]. Rocha and Clanton in "Blumgart’s Surgery of the Liver, Biliary Tract and Pancreas" expand on this by describing port placement of the camera, while Jackson and Evans delineate specimen bag use specifically in acute cholecystitis [6,7]. Together, these points highlight the need for a reproducible, teachable technique for gallbladder removal. 

Therefore, we provide clarity by including a detailed description, with clinical indications, for gallbladder removal without the use of a specimen bag. Additionally, our technique saves both time and money as it does not require a smaller scope to completely remove the gallbladder. As described in our technique, the large scope can remain in the umbilical port throughout the entire procedure. Contrary to current literature describing the need for a specimen bag, the surgeon has safely performed this technique in cases of acute cholecystitis [7].

Understanding the financial impact

The United States Centers for Disease Control and Prevention has developed criteria to define SSIs related to an operative procedure [8]. As one of the most common and expensive healthcare-associated infections, strategies to reduce SSI occurrence are beneficial financially and for the patient [9]. With gallbladder surgery being the most common elective procedure in the abdomen, further discussion is warranted regarding the need for specimen bags in these cases [10].

Most commercially available specimen retrieval devices are quoted at $30-$150 per disposable bag [11]. In a study of intraoperative costs, Gitelis et al. saw a 10% decrease in disposable instrument use per case [12]. A total savings of $1500 was seen when surgeons decided to not use a specimen bag at all. Considering the vast number of laparoscopic cholecystectomies performed each year in the United States, the savings to the health care system with reduced routine use of these devices could be astronomical. On the other hand, when the use of a bag is warranted, efforts are underway to create cost-effective or self-made disposable bags for surgical procedures [11].

SSIs and disposable bags in elective cholecystectomies

SSIs occur in 2.4%-3.2% of patients undergoing elective laparoscopic gallbladder removal [13]. Research evaluating these surgeries has found not only is the use of a specimen bag insignificant, but prophylactic antibiotics may be an unnecessary measure as well for SSI prevention [13,14]. The lack of efficacy found amongst these preventive measures could suggest that most port site infections do not result from direct contact of the gallbladder and wound. This is reinforced by the lack of correlation between typical biliary bacteria and wound infection cultures reported amongst elective cases [15,16].

Longer operating times are associated with an increased risk of SSI, as reported by Korol et al. [17]. The operating surgeon performing this technique hypothesized that his operating time decreased after removing this equipment from his surgical field. Without the use of the bag, another port is available to the surgeon and repositioning between ports is decreased. Future research is needed to explore the relationship between decreased operating time and SSI risk in elective cholecystectomies. Surgical time is likely influenced by confounding variables, such as increased complexity of the surgery itself.

In general, SSI rates following laparoscopic cholecystectomy are lower compared with open cholecystectomy [18]. This could be attributed to the shorter operating times and smaller incision sites seen in laparoscopic surgeries. Open procedures are usually saved for more complex cases where the risk for complication, including infection, is often increased. Laparoscopic techniques additionally hold a risk of complication that requires transitioning to an open procedure.

Additional risk considerations in laparoscopic surgery

Bleeding, abscess, bile leak, biliary injury, and bowel injury are the more serious complications that arise specifically during laparoscopic surgeries [19]. These risks could discourage surgeons from attempting unfamiliar techniques such as ours. Procedure reports have shown an exponential learning curve in laparoscopic skill amongst surgeons, with more experienced surgeons having the lowest complication rates [20]. The performing surgeon’s level of experience therefore should be considered prior to adopting a new technique.

## Conclusions

In this report, we describe an alternative approach to gallbladder removal in laparoscopic cholecystectomies that does not require the use of a specimen retrieval bag. Many surgeons report using disposable bags to decrease SSIs in elective cases, but research does not support this claim. Additionally, with a cost ranging from $30-$150 per unit, their use may be an unnecessary expense in elective procedures where retrieval bags are considered optional. Further research should be done to determine the effect of this technique on operating time and the incidence of SSIs in elective laparoscopic cholecystectomies. 
